# Platelet-Rich Plasma Intramuscular Injections — Antinociceptive Therapy in Myofascial Pain Within Masseter Muscles in Temporomandibular Disorders Patients: A Pilot Study

**DOI:** 10.3389/fneur.2019.00250

**Published:** 2019-03-19

**Authors:** Aleksandra Nitecka-Buchta, Karolina Walczynska-Dragon, Wojciech Michal Kempa, Stefan Baron

**Affiliations:** ^1^Department of Temporomandibular Disorders, Medical University of Silesia, Katowice, Poland; ^2^Institute of Mathematics, Silesian University of Technology, Gliwice, Poland

**Keywords:** platelet-rich plasma, myofascial pain, masseter muscle, intramuscular injection, muscle regeneration

## Abstract

**Background and Objective:** The objective of this study was to explore the nociceptive effect of platelet-rich plasma (PRP) intramuscular injections in myofascial pain of masseter muscles in patients with TMD.

**Methods:** Patients diagnosed with myofascial pain were assessed for eligibility for the study. Masticatory muscle disorder was diagnosed based on the Research Diagnostic Criteria for Temporomandibular Disorders (Ia and Ib). A total of 80 patients were enrolled in the study; 58 of them (21 male and 37 female, 29.4 ± 6.53 years old) met the inclusion criteria and were randomized to one of the two groups: Group I (*n* = 29) and Group II (*n* = 29). The first group received injections with PRP and the second group received injections with isotonic saline as the control group (0.9% NaCl). The Visual Analog Scale (VAS) was used to determine the pain intensity changes during follow-up visit**s** in each group.

**Results:** A significant improvement in pain intensity in VAS scale was observed, with 58% reduction in the experimental group and 10.38% in the control placebo group, 5 days after the injections (Day 5). The pain intensity reduction (VAS) 14 days after the injections (Day 14) in experimental group was 47.16 and 4.62% in control group, according to the baseline values (Day 0).

**Conclusions:** Intramuscular injection of PRP was a successful method for reducing myofascial pain within masseter muscles in temporomandibular disorders patients. However, the use of PRP for the treatment of myofascial pain within masticatory muscles requires further, clinical trials evaluation.

**Clinical Trial Registration:** Bioethical Commission at the Silesian Medical Chamber in Katowice, Poland 44/2017 as well as at ClinicalTrials.gov NCT03323567 (December 13, 2017).

## Introduction

Platelets are cytoplasmic fragments of megakaryocytes ~2 μm in diameter, which are formed in the human bone marrow. They produce adhesion molecules: fibrine, fibronectin, and vitronectin. Degranulation of platelets causes secretion and protein binding to target cells: osteoblasts, fibroblasts, and mesenchymal cells. As the result of cellular proliferation, synthesis of collagen, and production of extracellular matrix occurs. All products of degranulation are secreted approximately for 1 h (1). Dhurat et al. found that for optimal concentration of platelets of 1.25 × 106−1.5 × 106 per mL of PRP provides proliferation of endothelial cells and angiogenesis (1). There is an average level of 200,000 ± 75,000/μL blood platelet count in human blood (2). The therapeutic PRP counts up to 1 million platelets per 1 mL (1). Platelet concentration 2.5 times higher than in the whole blood concentration seems to be as effective as optimal platelet concentration (3). The goal of PRP in healing process is to concentrate the main growth factors from native blood and to reintroduce them in the injured tissue. Many different techniques are available for PRP preparation and it is difficult to get the same product with different protocols and technical conditions. The most popular and well-known form of blood-derived products for severe thrombopenia treatment is a concentrate for transfusion that contains 0.5 × 10^11^ platelets per unit (one unit is 1 dose for an adult, with 0.5 × 10^11^ platelets suspended in 45–65 ml of plasma) (3). PRP contains many growth factors such as: vascular endothelial growth factor, platelet-derived growth factor, and transforming growth factor-β1 (TGFβ-1). These are very important factors for angiogenesis, extracellular matrix changes, and cell production (3). PRP has been used in medicine since 1970s. Pihut et al. and Lin et al. have used them in the temporomandibular disorders (TMD) therapy (4, 5). Reurink et al. have used PRP in the therapy of skeletal muscles injuries (6). To the best of our knowledge there were no studies concerning intramuscular application of PRP in masticatory muscles.

Polish version of Research diagnostic criteria for temporomandibular disorders (RDC/TMD) was used in the study (7). Myofascial pain of masseter muscles can be a difficult issue for differential diagnosis in TMD patients. In most cases it is related to parafunctional activity during sleep, classified as sleep bruxism (8, 9). Bruxism leads to an excessive effort in masticatory muscles and consequently, to anaerobic metabolism and to muscle pain. According to Osiewicz et al. the frequency of muscle disorders in polish patients suffering from TMD was 56.9% (10). Different methods could be used for myofascial pain treatment as occlusal appliances, biofeedback or pharmacotherapy, but they are not always fully effective. Antinociception has a priority in the treatment of masticatory muscle disorder. The longer the muscle pain persists, the harder it is to overcome it. PRP intramuscular injections as a minimally invasive treatment is an additional therapy and can be used only in selected patients with myofascial pain, when other conservative methods do not bring relief.

Muscle regeneration and myogenesis are closely related to growth factors such as insulin-like growth factor-1, fibroblast growth factor-2, hepatocyte growth factor, transforming growth factor beta 1(TGFβ-1), tumor necrosis factor-α, platelet-derived growth factor, and prostaglandins. These factors stimulate proliferation, and differentiation of myoblasts (11). Hepatocyte growth factor activates satellite cells from which myoblasts develop. The level of TGFβ-1 and prostaglandins E-2 has to be balanced to prevent muscle fibrosis and scar tissue formation. PRP can not only promote muscle healing but also decrease pro-inflammatory and apoptotic cells, reducing inflammation (11, 12). PRP is a concentrate of these factors, it promotes muscle healing after intramuscular injection in painful muscles, but is also used in therapy of other diseases, such as: tendonitis, arthritis, osteoarthritis, wound healing, ophthalmology and tissue engineering.

The aim of this study was to explore the nociceptive effect of platelet-rich plasma (PRP) intramuscular injections in selected patients with myofascial pain of masseter muscles.

## Materials and Methods

### Study Participants

Eighty adult patients were selected from the population of subjects referred to the Department of Temporomandibular Disorders. Fifty nine subjects (38 female and 21 male, mean age 29.35 ± 6.61) suffering from myofascial pain of the masseter muscles were found eligible and enrolled to the study.

The inclusion criteria were:

1. Age ≥18 and ≤80.
2. Presence of myofascial pain within masseter muscles according to the RDC/TMD (Ia and Ib) (7).
3. Patient's agreement for participation in this study.

The exclusion criteria were:

1. Patients being treated with or addicted to analgesic drugs and/or drugs that affect muscle function.
2. Patients with neurological disorders, and/or neuropathic pain, and/or headache.
3. History of the head or neck trauma in preceding the enrollment 2 years.
4. Edentulous patients.
5. Patients after radiotherapy.
6. Presence of mental disorders.
7. Pregnancy or lactation.
8. Pain of dental origin.
9. Diagnosis of malignancy.
10. Drug and/or alcohol addiction.
11. Patients with needle phobia.

This study was approved by the Bioethical Commission at the Silesian Medical Chamber in Katowice, Poland (number 44/2017), and retrospectively registered at ClinicalTrials.gov (NCT03371888). The study was performed in accordance with the Declaration of Helsinki as well as the International Conference on Harmonization: Guidelines for Good Clinical Practice. All included patients gave their consent to participate in the study and received verbal and written information describing the trial.

### Study Protocol

**Figure 1:**
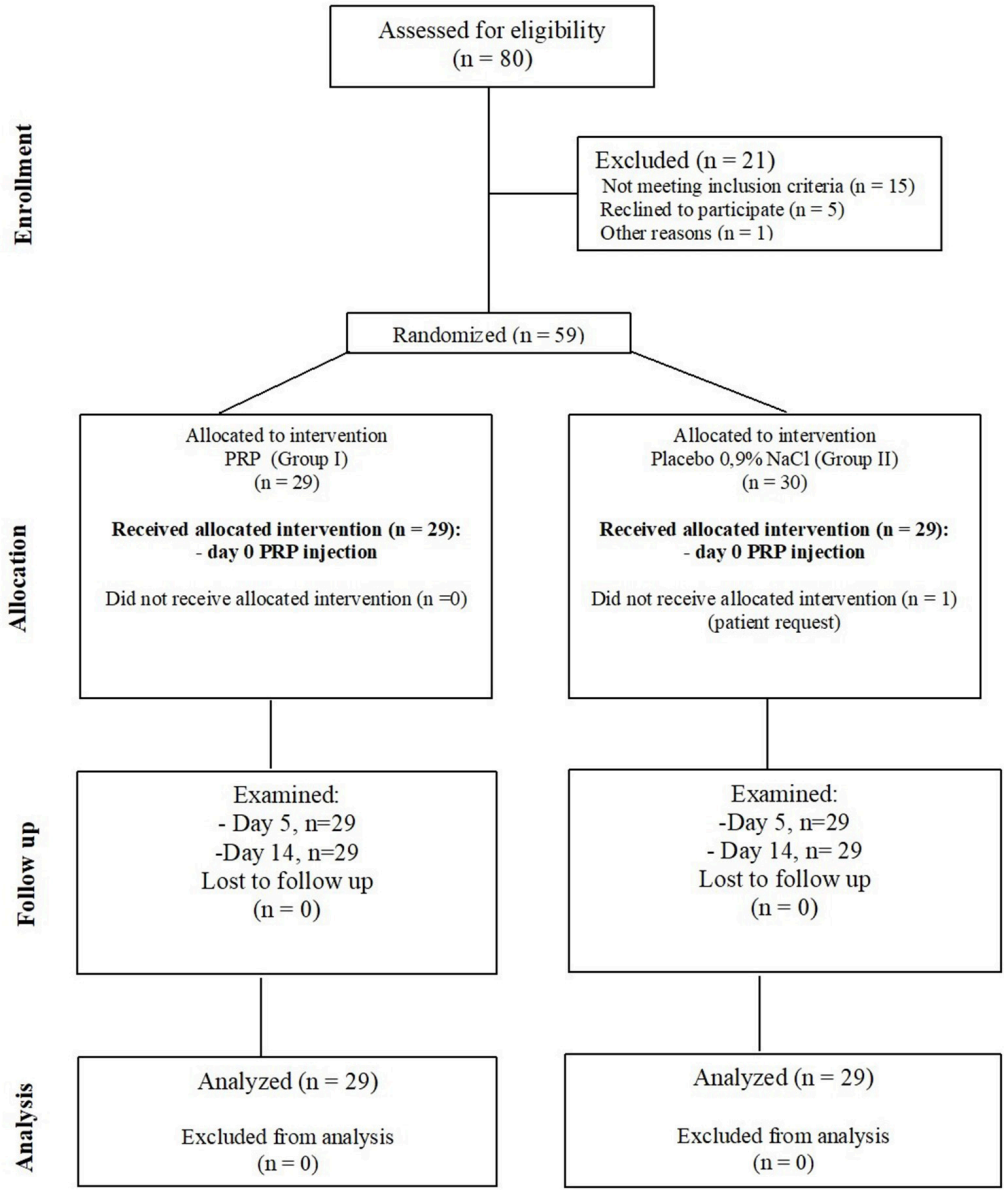
The two-arm diagram of the flow of participants in the study.

This randomized, controlled, double-blind, two-arm trial followed the consolidated standards of reporting trial statement (12) and was performed between December 7, 2017 and December 24, 2018 in the Department of TMD. The patients (*n* = 59), of both genders were randomized into one of two groups: experimental (Group I, *n* = 29), and control (Group II, *n* = 29) (Figure 1). Patients were randomized by choosing the number from a closed envelope. Groups were structured as follows: Group I: *n* = 29, 17 female, 12 male, mean age 28.9 ± 6.23 years and Group II: *n* = 29, 20 female, 9 male, mean age 29.8 ± 6.99 years. Patients were blinded to the substance injected during the procedure. Only the study coordinator, knew what substance was prepared in the disposable syringe. Another research team member was not informed to which group the patients were allocated during the follow-up visits (Day 5 and Day 14) while checking the pain level in Visual Analog Scale (VAS). PRP was prepared for both groups: I and II before the injection on Day 0. PRP in controls was prepared and frozen at −20°C for the future use (13).

Pain intensity was measured with VAS scale (0 = no pain, 10 = the worst pain that one can imagine) before (Day 0), during (Day 5), and after (Day 14) of the therapy with PRP injections.

The trial consisted of three visits:

1. Baseline visit: injection of study substances—Day 0
2. Control no. 1 after 5 days–Day 5
3. Control no. 2 after 14 days–Day 14

The activities undertaken by the investigators during the trial are presented in Table 1.

**Table 1:** Activities of investigators during the trial Visual Analog Scale (VAS I.1, VAS, Group I, first measurement, Day 0).

**Visit**	**1 (Screening and inclusion)**	**2 (Baseline)**	**3 (Control 1)**	**4 (Control 2)**
Day of the study	–	Day 0	Day 5	Day 14
Injection PRP or Placebo	–	+	–	–
Measure VAS	–	+	+	+

### PRP Preparation Protocol

Approximately 40 mL of venous blood was harvested from the cubital vein in four anticoagulant vacutainer tubes (Vacuette 9 mL, sodium citrate 3.2%, Greiner Bio-One, Austria), with a dedicated large-bore needle (butterfly valve fitted to a syringe with long adapter BD Vacutainer Safety-Lok blood collection set with pre-attached holder 21G, 19 mm). The blood was mixed (5 times to prevent micro bunches creation) with an anticoagulant (3.2% sodium citrate). Pure-PRP was prepared as described by Ehrenfest (3). Manual PRP protocol with double spin centrifugation process was used with the centrifuge Zenithlab80–2C. First step of centrifugation: a “soft” spin was performed with an anticoagulant at 1,500 rpm for 5 min (14, 15). Three typical layers of whole blood were found: red blood cells, platelet poor plasma, and a PRP layer between them. Platelet poor plasma and PRP were collected as supernatants over the red blood cells from the tube and transferred into another sterile tube. The temperature during centrifugation was room temperature: 21°C. The second step was a “hard” spin at 3,200 rpm for 15 min. In this process about 6 mL of pure-PRP was obtained. There were no leucocytes or low-density fibrine network in the produced PRP. There was no blood chilling before centrifugation and blood was immediately processed with a low force.

### Treatment

During the intervention, painful muscle parts within the masseter muscles were identified with palpation of the masseter muscle and in each group the same amount of the appropriate substance was injected. In all groups, disposable syringes (5 mL) and needles (BD Microlance, 0.3 × 13 mm) were used for injections. Group I PRP and in Group II isotonic saline (0.9% NaCl) were injected bilaterally into the right and left masseter muscles at 3 painful points at each site (6 × 0.5 mL = 3 mL) near the origin, under the zygomatic arch. Injections were deposited 0.5–1.0 cm under the skin surface.

### Treatment Outcome Measures

For measuring a treatment outcome, VAS scale was used at Day 5 and Day 14 follow-up visits.

### Sample Size Estimation

The minimum sample size necessary to achieve the presumed accuracy of the estimation is determined by the two-stage Stein method.

### Statistical Analysis

For the statistical analysis the Statistica software, version 13.1. by Statsoft Polska was used.

To demonstrate the effect of the applied treatment on the level of pain, the following parametric tests were used for two independent tests (experimental group, Group I and control group, Group II:

- *t*-test for two means;
- tests for two variances (F test, Levene test, and Brown-Forsythe test).

In the *t*-Student test, the null (test) hypothesis H_0_ was the equality of the corresponding means in the experimental Group I and the control Group II; for variance tests, it was the equality of the corresponding variances.

At the end, we will verify the null hypothesis about the equality of the distribution of pain levels in both groups of patients, using

- Wald-Wolfowitz runs test;
- U Mann-Whitney test.

## Results

**Figure 2:**
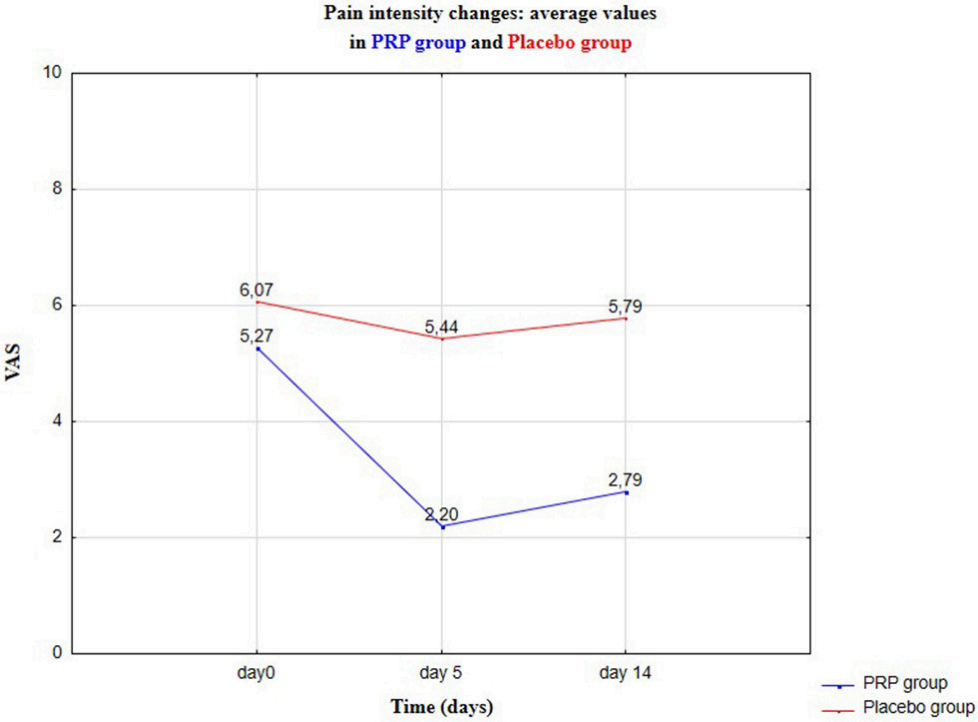
Pain intensity changes in VAS—average values: baseline visit (Day 0), control 1 (Day 5), and control 2 (Day 14).

**Figure 3:**
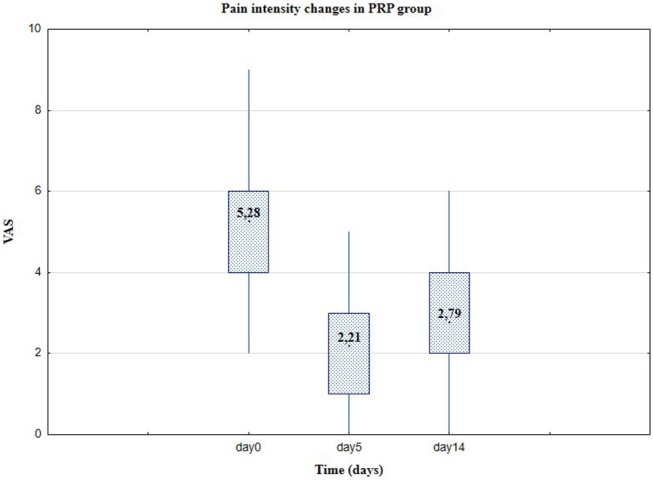
Pain level in VAS, Group I-platelet-rich plasma (Day 0, 5, and 14).

**Figure 4:**
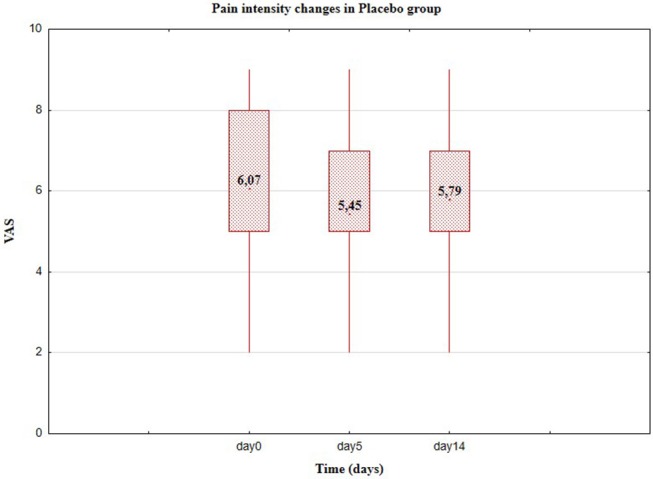
Pain level in VAS, Group II-placebo (Day 0, 5, and 14).

There were no statistically significant differences in age or gender between the groups (*p* > 0.05) (Table 2). There was a 58% reduction in pain intensity in Group I, 5 Days after PRP injection in masseter muscles. In the control group II, after isotonic saline injection, there was 10.24% reduction in pain intensity (Figures 2–4).

**Table 2:** Baseline characteristics of 58 patients included in the study.

	**Group I**	**Group II**
*n* Male/Female	12/17	9/20
Age (years)	28.9 ± 6.23	29.8 ± 6.99

### Descriptive Measures and Confidence Intervals

In the case of the experimental group I (PRP), the values of key descriptive statistics and the limits of confidence intervals for the mean and standard deviation of pain level at the confidence level of 0.95 (or 95%) were as in Table 3. In the case of PRP application, after 5 days, the pain decreased substantially (considering the average level of the variables, from ~5.28 to ~2.21, average reduction 58.15%). After 2 weeks (Day 14), the average pain level increased slightly (to ~2.79, average reduction 47.16%). It is worth considering a significant decrease in the median of the examined feature: from the value of 5 to the value of 2 at Day 5. Thus, immediately after PRP application, 50% of patients experienced pain at the level of 5 or higher; after 5 days, 50% of patients experienced pain at the level of at least 2, but at the same time at 50% at the level of at most 2. An increase in the coefficient of variability in subsequent follow-up tests is characteristic. This is due to a decrease in the average level of pain with only a slight change in the standard deviation: therefore, the average level of pain is significantly reduced, but the degree of dispersion of the results is not significantly changed (differences in the level of symptoms in different patients).

**Table 3:** Descriptive measures and confidence intervals in experimental group (Group I).

**Characteristic**	**Day 0 (Baseline)**	**Day 5 (Control 1)**	**Day 14 (Control 2)**
Mean	5.27	2.20	2.79
Median	5.00	2.00	3.00
Range	7.00	7.00	6.00
SD	1.79	1.56	1.69
CV	33.94%	71%	60.80%
Confidence interval for mean (95%)	(4.59, 5.95)	(1.61, 2.80)	(2.14, 3.43)
Confidence interval for SD (95%)	(1.42)	(1.24)	(1.34)

For control experiments, the confidence intervals for the mean and standard deviation at the confidence level 1–α = 0.95 were constructed.

Relevant results for Group I are represented in Table 4. The average level of pain does not change significantly, also the standard deviation remains at almost the same level; similarly for median, range, and coefficient of variation. Pain reduction in the control Group II was observed from the average level of the variables: from ~6.07 to ~5.44 (reduction 10.38%) after 5 days and after 14 days to ~5.79 (reduction to 4.62%). Compared with the results in Table 2, this indicates a significant effect of the PRP therapy, on the pain level of patients. Pain levels in VAS in the experimental (Group I) and control (Group II) are presented in Figures 2, 3, respectively.

**Table 4:** Descriptive measures and confidence intervals in the control group (Group II).

**Characteristic**	**Day 0 (Baseline)**	**Day 5 (Control 1)**	**Day 14 (Control 2)**
Mean	6.06	5.44	5.79
Median	6.00	6.00	6.00
Range	7.00	8.00	7.00
SD	2.01	2.14	1.93
CV	33.22%	39.42%	33.38%
Confidence interval for mean (95%)	(5.30, 6.83)	(4.63, 6.26)	(5.05, 6.52)
Confidence interval for SD (95%)	(1.60, 2.72)	(1.70, 2.90)	(1.53, 2.61)

### Parametric Tests

For both control visits: after 5 days and after 14 days, we have rejected the null hypothesis about the equality of the average level of pain in the experimental and control groups. The tests confirm the earlier observation that the level of pain in the experimental group, Group I, is significantly lower than the corresponding level in the control group, Group II, both after 5 and after 14 days. The tests results are given in Table 5.

**Table 5:** Parametric tests results for the baseline, control 1, control 2 visit.

	**Day 0 (Baseline)**	**Day 5 (Control 1)**	**Day 14 (Control 2)**
**Test**	***p*****-value**	***p*****-value**	***p*****-value**
T	0.11	0.00	0.00
F	0.53	0.10	0.49
Levene	0.47	0.05	0.84
Brown-Forsythe	0.47	0.06	1.00

### Non-parametric Tests

Non-parametric tests confirm the thesis that the level of pain in Group I is significantly lower than the analogous level in Group II, both after 5 and after 14 days. The results are given in Table 6.

**Table 6:** Non-parametric tests results for the baseline, control 1, control 2 visit.

	**Day 0 (Baseline)**	**Day 5 (Control 1)**	**Day 14 (Control 2)**
**Test**	***p*****-value**	***p*****-value**	***p*****-value**
Wald-Wolfowitz	0.50	0.00	0.00
U Mann-Whitney	0.09	0.00	0.00

### Adverse Effects

After the injection of PRP or isotonic saline in the masseter muscle, three patients in Group I, and one patient in Group II, reported edema and muscle pain. Seven patients had an adverse side effect: bruising, as a result of blood harvesting procedure from the blood vessel. These symptoms were only temporary and completely reversible. There were no serious adverse effects during the trial.

## Discussion

The 58% reduction in pain intensity, 5 days after PRP injection in masseter muscles was achieved, comparing to the control group, where the 10.24% reduction in pain intensity was observed. An intramuscular administration of PRP is being used more frequently as a popular treatment for skeletal muscle injuries in athletes (5, 15). Better healing effects of muscle injuries after intramuscular injections are observed and potential benefits of PRP in myofascial pain treatment have been demonstrated in many studies but these studies are not related to orofacial muscle pain. Most studies analyze the impact of PRP intra-articular injections on the function and condition of the temporomandibular joint (4, 5).

In patients suffering from TMD it is important to stop the pain in the first place and after pain relief other types of therapies should be included, such as treatment with intraoral occlusal appliances, anti-inflammatory treatment, and muscle tension-pharmacotherapy, psychotherapy: parafunction prevention, and treatment of bruxism (9).

The use of PRP is an innovative method. It carries almost no risk of complications and although not all authors agree with its high effectiveness of action, according to this research study it is an effective treatment for masseter muscle myofascial pain (5, 16, 17). Martinez-Zapata et al. in his clinical trial obtained a shortening of healing time from 38 days in the control group to 31 days in the study group with PRP intramuscular injection (18). In addition, he also obtained fewer relapses: 7 people in the control group and only 1 person in the study group. He did not find any significant improvement in the duration of healing. In the case of masseter muscle myofascial pain, the possibility of obtaining such results would be a very promising treatment method.

In Franchini meta-analysis, the author has proved the lack of effectiveness of the PRP in orthopedics (16). According to the authors of the mentioned study, the therapeutic effect is clear, however short-term (up to 14 days). Based on the literature data, the best muscle healing was observed 2–10 days after injection (19), probably because of the platelet half-life time, which *in vivo* is ~7–10 days (2). The effect of PRP found in this study is not long-lasting and the injections should be repeated, more or less after 14 days, when the level of pain is slightly increased. Hammond et al. reported a significant functional improvement in muscle function at Day 3 to Day 14 after intramuscular injection of PRP in rats (20). According to the authors, PRP injections in masseter muscles should be repeated until a satisfactory therapeutic effect is obtained, often as a supportive treatment for other therapies used in TMD. Ineffective therapies using PRP may result from different protocols of PRP preparation, differences in the methodology of administration, and specificity of the disease entity. Intramuscular injection of PRP into masseter muscles in myofascial pain resulted in best antinociceptive results. The pain level reduction in placebo Group II, was probably due to therapeutic injections of isotonic saline, to some extent similar to acupuncture. Despite the satisfactory results and an innovative contribution to myofascial pain research, this study has some limitations: a small study group and a short follow-up observation.

## Conclusions

In selected patients with TMD, suffering from myofascial pain, the intramuscular injection of PRP could be considered as additional, successful therapy in pain relief, when other conservative methods do not bring relief. The further investigation on safety and efficacy of the method are needed.

## Data Availability

The datasets supporting the conclusions of this article are included within the article. Access to these data will be considered by the corresponding author upon request.

## Author Contributions

AN-B created trial concept, performed intramuscular injections of PRP and wrote and edited the manuscript. KW-D collected information concerning pain intensity changes using VAS. SB conducted the randomization and edited the manuscript. Statistical calculations were carried out by WK. All authors read and approved the final manuscript.

### Conflict of Interest Statement

The authors declare that the research was conducted in the absence of any commercial or financial relationships that could be construed as a potential conflict of interest.
